# Scaling up food pricing policies in the Pacific: a guide to action

**DOI:** 10.1136/bmjgh-2023-012041

**Published:** 2023-10-09

**Authors:** Erica Reeve, Amerita Ravuvu, Ellen Johnson, Selai Nasiga, Tom Brewer, Sarah Mounsey, Anne Marie Thow

**Affiliations:** 1 Global Centre for Preventive Health and Nutrition (GLOBE), Institute for Health Transformation, School of Health and Social Development, Deakin University, Geelong, Victoria, Australia; 2 Non-Communicable Disease (NCD) Prevention and Control Programme, Public Health Division, Pacific Community, Suva, Fiji; 3 Sydney School of Public Health, The University of Sydney Menzies Centre for Health Policy, Sydney, New South Wales, Australia; 4 Independent consultant, Commonwealth Secretariat, London, UK; 5 Australian National Centre for Ocean Resources and Security, University of Wollongong, Wollongong, New South Wales, Australia

**Keywords:** Health policy, Nutrition

## Abstract

There are calls for governments around the world to adopt pricing policies, including taxes, subsidies and price controls that ensure all people have access to, and can afford, healthy diets. Despite the strong potential of pricing policies to promote healthy diets and to support a post-COVID-19 recovery, there are gaps in evidence with regard to ‘how’ to design and apply effective food taxes in practice, and countries report challenges in navigating the different policy options.

In this practice piece, we examine the global evidence for food taxes with a view to identifying practical lessons for policy design, adoption and implementation, using the Pacific Islands Region as a case study. We present a systematic resource that draws on locally generated evidence, and a Pacific conceptualisation of healthy diets, to address considerations in setting the tax base, rate and mechanisms, and to ensure tax targets are clearly identifiable within national tax and administrative systems. Health and Finance collaboration at the country level could ensure tax design addresses concerns for the impacts of food taxes on employment, economics and equity, as well as position food taxes as an opportunity to fund revenue shortfalls faced by governments following the COVID-19 pandemic. We demonstrate a need to review other policies for consistency with national health objectives to ensure that countries avoid inadvertently undermining health taxes, for example, by ensuring that foods with known non-communicable disease risk are not being price protected or promoted.

**Summary box**

- Pricing policies create incentives within food systems and can be used to reduce consumption of products that pose negative societal effects.
- Food taxes are recognised as a critical revenue opportunity in a post-COVID-19 recovery, but lack of cohesive evidence on ‘how’ to establish food taxes and subsidies has been a key barrier for countries.
- This practice piece offers a framework for stepping through considerations for food taxation, including how to nuance these to country context, using the Pacific Island Countries as an example.
- We present options for defining and identifying the objects of taxation that account for broader health and environmental implications, and recommend that taxes comply with existing tax systems, and are set at a rate of 20% of more, to improve feasibility and impact.
- It is recommended that countries review other food pricing policies (including import excises, subsidies and price controls) for consistency with health and environmental objectives. Accompanying food taxes with subsidies on healthier alternatives with a lower environmental impact will make food taxes more equitable and impactful.

## Introduction

Greater production and affordability of processed foods that are high in sodium, fat and sugar has played a central role in the development of non-communicable diseases (NCDs), which are now responsible for over 70% of global mortality.^1^ In addition, the production of these foods is a known contributor to climate change, environmental degradation and biodiversity loss globally.^2^ Policy-makers and researchers are examining policies that shift eating patterns such that they promote both social, environmental and health outcomes,^3^ but they also have significant concerns related to record-high food prices, owing to major food chain disruptions linked to the COVID-19 pandemic and other recent crises.^4^

Pricing policies create incentives within food systems and can be used to reduce consumption of products that pose negative societal effects or to increase consumption of net positive foods.^5^ There are calls for governments around the world to adopt pricing policies, including taxes, subsidies and price controls, that ensure all people have access to, and can afford, healthy diets.^4 6 7^ Pricing policies are recommended by the WHO to improve relative affordability of healthy foods compared with less healthy alternatives, for the prevention of NCDs.^7^ Governments have a long history of taxing and subsidising food and agricultural inputs, and there are calls for tools that better reflect the negative externality of food production,^8–10^ by incentivising production and consumption of more sustainable food options alongside other proposed supply or market-based approaches.^3 4 10^

Pricing tools have proved impactful in reducing consumption of sugar-sweetened beverages (SSBs) and tobacco,^11^ and there is some evidence that food taxes and subsidies may reduce unhealthy consumption and associated morbidity.^12 13^ Food taxes also send important ‘signals’ to consumers and producers that the government is serious about addressing unhealthy diets, and to reconsider those foods and beverages that are the target.^14^ Despite the strong potential of pricing policies to promote healthy diets, there are gaps in evidence with regard to ‘how’ to design and apply effective food taxes in practice.^6 15^

Pacific Islands Countries (PICs) were early adopters of SSB taxes.^16 17^ They are now leading global action on NCD prevention by exploring taxes on foods high in sodium, sugar and fat (online supplemental material 1), to address persistent high rates of diet-related NCDs,^18^ childhood obesity^19^ and micronutrient deficiencies.^18^ Food taxes are also recognised as a critical revenue opportunity in a post-COVID-19 recovery, with PICs having just experienced the largest economic contraction in four decades.^20^

10.1136/bmjgh-2023-012041.supp1 Supplementary data

However, food taxes and subsidies can be complicated to design and administer,^21^ including in PICs.^15 22^ Major points that countries must address include documenting relevant consumption and social impacts, in addition to meeting other requirements of policy process,^21^ often in the face of strong opposition from food industry.^23^

In this paper, we adapt a framework from Thow *et al* 21 to provide specific guidance for countries considering adopting or expanding food taxes for NCD prevention (table 1). We examine the global evidence and develop policy guidance for the design, advocacy and implementation of food pricing policies, and consider how these might be addressed, using the Pacific context as an example. Evidence was sourced from WHO and Pacific Community policy guidance documents, country case studies and other research emerging in this space. Considerations were informed through engagement with policy partners from regional technical support organisations The Pacific Community and the WHO Division of Pacific Technical Support. Although there are unique features of the Pacific context, previous research has indicated that processes and politics for taxation are widely relevant globally.^17^

**Table 1:** Framework of considerations for food and beverage pricing policies

Considerations for implementation	Feasibility in the Pacific Islands context
Objects of taxation	Defining the targets of food and beverage taxation (NCD risk and environments)
	Demonstrating problematic consumption of target foods
	Administrative considerations for the implementation of food and beverage taxation
Setting the rate and mechanisms	Determining rates that will impact consumption
	Mechanisms for applying taxation
Navigating fiscal and political dynamics	Improving political palatability of food taxation Revenue benefit Industry and employment Equity concerns
	Creating a coherent policy environment complementary to policy objectives
Framework adapted from Thow *et al* 21 by authors	

NCD, non-communicable disease.

## Consideration 1: objects of taxation

### Global evidence: defining foods that are the targets of taxation

First, there is a need to adopt a transparent system for determining which foods will, or will not, be subject to a tax, particularly with regard to which foods are deemed as carrying a heavy social cost.^24^ The effects of a food item on NCD risk or the environment, together with the price responsiveness, are key determinants for overall impacts of food taxes.^25^ A key challenge for the adoption of food taxes has been demonstrating a convincing link between specific foods or groups of food commodities and diet-related NCD risk or environmental outcomes,^10 26^ such that a tax would create meaningful incentives from a health or environmental perspective.

Overcoming these challenges requires a system for classifying food for policy purposes, which also takes into account broader health and environmental implications.^26–28^ The main dimensions include: (1) nutrients associated with risk, (2) national dietary guidelines, (3) foods containing ‘unhealthy’ ingredients (4) level of processing to produce the food or (5) potential food systems impacts (table 2). One important consideration is that the criteria against which a tax is applied can incentivise industry and consumer change. For instance, applying a tax against nutrient thresholds (eg, sodium thresholds) can encourage reformulation or substitution of food products containing high levels of sodium, to below a given threshold. Applying a tax against specific ingredients, whether it be for health or environmental reasons, may encourage substitution to different ingredients. Countries may choose to combine elements of these approaches, but a simpler evidence-based approach will be more straightforward to rationalise and to implement.

**Table 2:** Methods of defining unhealthy or unsustainable food for taxation

Method	Application for food taxation and subsidy
Nutrient profiling	Classifies food by establishing nutrient thresholds above (or below) which a policy restriction is applied, most often based on a product’s total energy, sugar, fat, saturated fat and/or sodium contents.^40^ Currently used to underpin restrictions on marketing, school food guidance and pricing policies.^41^ Nutrient profiling models are considered to be non-discriminatory and transparent, and they incentivise reformulation by companies. However, lack of consistency across models^26 42^ has created uncertainty for policy advocates, leaving space for criticism by policy opposition.
Healthy or unhealthy food guidelines	Classifies food into core groups, and in many settings this includes a set of foods to limit.^43^ Evidence suggested that food group or commodity-specific taxes may not be as impactful as nutrient-based taxes (eg, nutrient profiles).^34 44^ However, food group-based subsidies have been found to be impactful in the case of fruit and vegetables.^11^
Ingredient-based taxation	Classifies food as unhealthy based on specific ingredients with a demonstrated relationship with NCDs (specifically sugar, saturated fat, trans fatty acids, sodium). For instance, dairy foods containing ‘added sugars’ (eg, sweetened milk or yoghurt) or snack foods containing ‘hydrogenated vegetable oil’.
Ultra-processed food	Classifies food based on their degree of refinement, and the number of ingredients added, during processing, as a proxy for unhealthy food,^45^ including for taxation.^46^ Evidence is mounting that ultra-processed foods also carry a higher negative environmental externality,^47^ and their reduction should be central to the protection of biodiversity.^48^ However, existing systems are thought to be more prone to misclassification^49^.
Foods with negative food systems impacts	Classifies food on the basis of negative environmental externalities, including greenhouse gas emissions, water use,^9 26^ cropland use, nitrogen use, phosphorus use, consumptive freshwater use and terrestrial extinction rate.^9 50^ Some high-income countries have taxed red-meat as a way of promoting plant-based diets.^8^ More recently, researchers have developed an ‘unsustainable food database’ that factors greenhouse gas emissions, land use, water stress, and eutrophication potential to profile 57 000 food items based on their ingredients.^51^

NCD, non-communicable disease.

In considering the incentives for behaviour change that result from taxation, it is also important to consider the price elasticity of foods. Applying a tax to food(s) with a high price elasticity is more likely to generate a change in consumption (as seen with SSBs), whereas taxing foods with low price elasticity will simply raise revenue.^5^ Foods with a large number of substitutions will have a higher price elasticity (ie, more responsive), suggesting a need to design taxes that include all likely (undesirable) substitutions.^5^ Price elasticities have not yet been developed for use in low- and middle-income countries (LMICs).^11^

### Pacific context: defining foods that are the targets of taxation

Pacific Island governments have already begun adopting product-specific subsidies and taxes based on demonstrated (eg, high volume) or perceived health implications (eg, ‘fatty’ or ‘healthy’). For instance, Fiji increased import duties on palm oil from 15% to 32%, as a result of a 10-fold increase in palm oil imports between 2000 and 2009, in response to emerging evidence surrounding the links between hydrogenated vegetable fats and NCD risk.^29^ The Government of Tonga adopted a tax on lard/dripping, SSB and imported fatty meats in response to concerns around growing obesity risk.^30^

A nutrient-based criteria for taxation has the benefit of incentivising food reformulation by manufacturers seeking to avoid a tax being applied to their products. In the Pacific, canned fish makes a substantial contribution to dietary protein intake, and canneries are an important value-added industry. However, canned fish is also a significant contributor to sodium intake in PICs.^31^ Sodium thresholds have already been agreed in the Pacific for processed meat and canned fish.^31^ Establishing a nutrient-based tax against the sodium content of all processed meat, fish and poultry products could incentivise local manufacturers to reformulate. Applying thresholds to both imported and locally manufactured processed meats would eliminate concerns regarding trade discrimination.^24^

A food-based criteria for taxation could be adopted in alignment with the Pacific Guidelines for Healthy Living (2018) (box 1). These guidelines represent a Pacific conceptualisation of a healthy diet, emphasising locally cultivated and traditional foods as ‘foods to choose’ across three core food groups (box 2). A food-based criteria could be strengthened with studies demonstrating a convincing link between increasing consumption of ‘foods to avoid’ and rising rates of diet-related NCDs, as has been done in Samoa, the Solomon Islands and Tonga.^15 24 32^

Box 1 **Pacific guidelines for healthy living (2018)**

Foods to choose

- Energy foods: local root crop, tree-grown starches (eg, banana), whole grain breads.
- Body building foods: fish, eggs, local lean cuts of chicken and meat, nuts, beans and legumes, reduced fat milk and milk products.
- Protective foods: all fresh and frozen fruit and vegetable, locally grown.

Foods to avoid

- Energy foods: processed and deep-fried foods, sugar and free sugars, sugar-sweetened beverages.
- Body building foods: processed meat (spam, canned corned meats, burgers, sausages), condensed milk, mutton flaps.
- Protective foods: canned vegetables, dried fruits with added sugar or preservatives, cordials and fruit drink, fruit juice and fruit juice concentrate.

High salt foods are those with >2 g/100 g of food.

Choose foods with <2 g/100 g saturated fat.

Choose foods with <10 g/100 g sugar.

Pacific guidelines for a healthy living: a Handbook for health professionals and educators (2018). The public health division of the Pacific community, new Caledonia

Box 2 **Policy support for food taxation in Pacific Islands Countries**

- Cook Islands Non-Communicable Disease Risk Factors STEPS Report 2013–2015: ‘…explore use of taxes to regulate consumption’.
- Fiji Non-Communicable Diseases Strategic Plan 2015–2019: ‘Pursue share of taxes from revenue on…‘unhealthy’ food for health promotion’.
- Kiribati Health Strategic Plan 2016–2019: ‘Investigate alternative sources of revenue for the health sector, such as directing a proportion excise tax on tobacco, alcohol and unhealthy foods directly to the health budget’.
- Prevention of Non-Communicable Diseases: Nauru Strategy Action Plan 2018–2020: ‘Put in place mechanisms for economic incentives including taxes and subsidies that encourage healthy choices for food’.
- Samoa National Noncommunicable Disease Control Policy 2018–2023: ‘Increase excise tax on sugary drinks, salty foods and foods high in fat’.
- Solomon Island’s Multi-sectoral National Noncommunicable Disease Strategic Plan 2019–2023: ‘…reduce consumption of local and imported food and drink products that are high in sugar, salt and fat content…through targeted preventative measures, taxes, and better regulation’.
- Tonga National Strategy for Prevention and Control of Non-Communicable Diseases 2015–2020: ‘Economic measures increase the cost of less healthy foods and reduce the cost of locally produced healthier foods’.
- Vanuatu Ministry of Health Sector Strategy 2017–2020: ‘…broadening of ‘sin taxes’—taxation on sugary drinks and other unhealthy foods’.

Information sourced by authors by reviewing national health and finance policies across 22 countries (online supplemental file 1).

As an alternative, a hybrid model that combines a food-based dietary guidelines approach with nutrient thresholds could strengthen the basis for taxing unhealthy foods. The Pacific Guidelines for Healthy Living and the Pacific Salt Reduction Targets each identify nutrient thresholds across a limited number of food categories, which could form a convincing basis for a hybrid model.

Taxes for promoting environmental sustainability (table 2) generally draw on a range of information sources (eg, point of origin, food composition, greenhouse gas emissions and water use). However, a lack of data on these characteristics may form a barrier to their implementation in PICs. Foods characterised as ‘foods to choose’ in the Pacific Guide are well in alignment with global recommendations for healthy and sustainable diets, because they make a clear delineation between minimally processed foods and highly processed and packaged alternatives, and promote the consumption of locally available and traditional foods, including non-haem sources of iron and protein (eg, fish, poultry, tree nuts and eggs) (box 1).

### Global evidence: identifying levels of consumption of ‘problem’ foods to guide tax design

The second stage in selecting the objects for taxation, once taxable foods have been defined, is to identify which ones are being consumed in amounts that are likely to contribute to diet-related NCDs or environmental degradation.^32^ A potential source of data for this are household expenditure surveys, which are collected in some form by more than 120 countries. Household expenditure surveys provide data on purchases rather than dietary intake, thereby giving additional insights into consumption of foods (potentially) subject to taxation—for example, foods that would appear only as/within composite foods in a nutrition survey. This can help to differentiate between foods purchased as processed (eg, a takeaway burger) and the same foods prepared at home (reflected by purchases of bread, meat, etc)—an important nutritional distinction in cases where only one may be taxed. Data from household expenditure surveys can disaggregate against income quartiles and urban/rural status, providing greater clarity on the nature of the problem, including who will be paying the tax or benefit from the tax.^32^

### Pacific context: identifying levels of consumption of ‘problem’ foods to guide tax design

**Figure 1:**
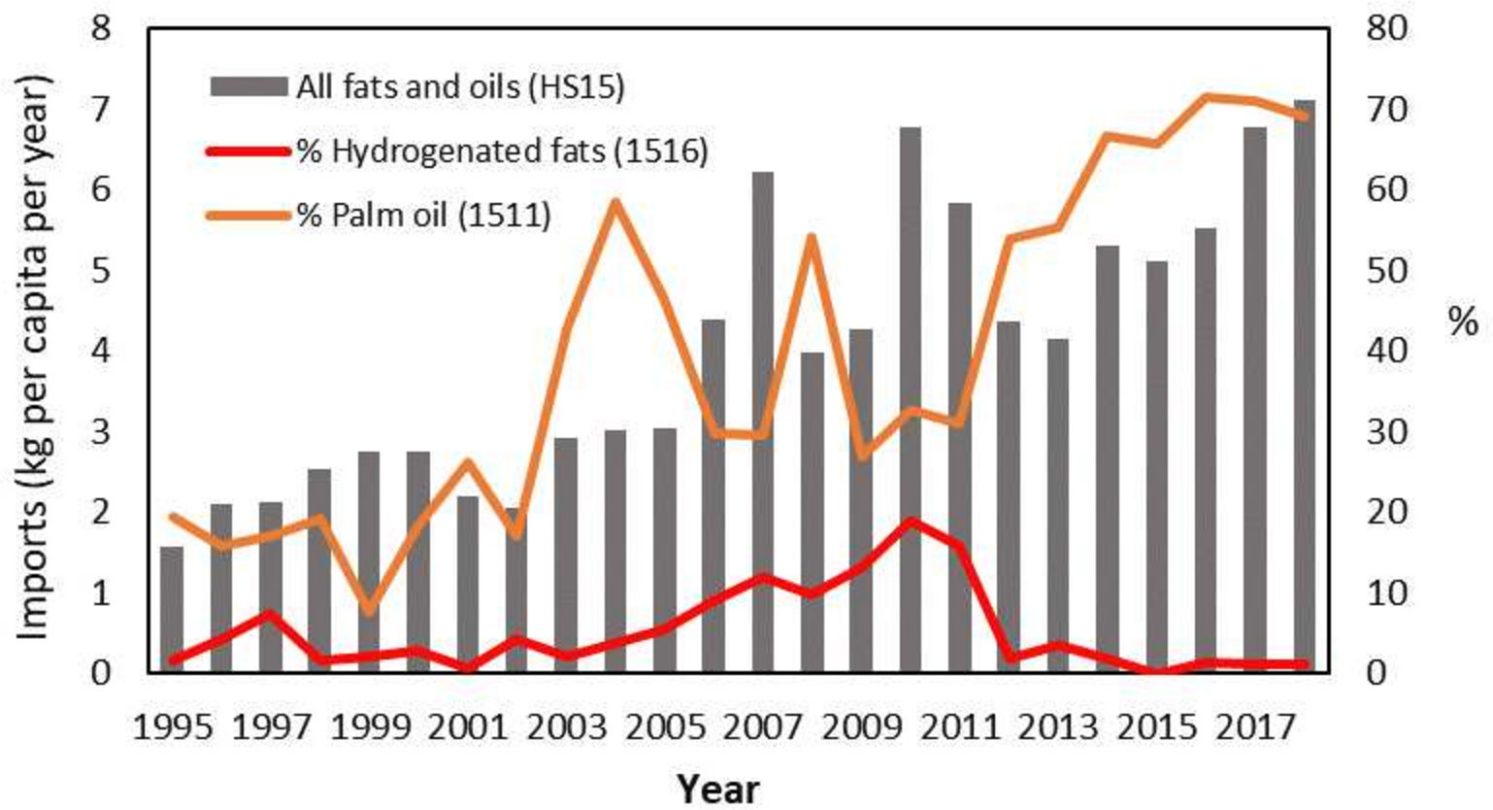
Illustrative example, using Vanuatu imports, of variation in differences in quantity of commodities traded at different levels of HS aggregation. Imports of all fats and oils increase through time while different types of fats and oils, which drive different health and environmental outcomes, vary in their quantity through time. Data from Brewer *et al*.^33^ HS, Harmonised Tariff Systems.

Lack of dietary intake data has previously been a key barrier for Pacific countries seeking to adopt evidence-based food environment policies.^15^ However, work is underway in the Pacific to translate dietary intake from household expenditure surveys (undertaken every 5–10 years) into meaningful indicators of consumption for countries.^32^ In addition, ass import dependent countries, a new Pacific Food Trade Database (PFTD) can provide insights on food and beverage consumption trends in each Pacific country over time.^33^ Figure 1, derived from the PFTD, demonstrates an increase to the proportion of imported cooking oils (HS15) over time, with detail on hydrogenated vegetable fats (1516) and palm oils (1511) (which are high in saturated and/or trans fatty acids) in Vanuatu (1995–2017). These reflect a greater than fourfold increase in oil imports over 20 years, demonstrating an opportunity for governments to tax specific cooking oils to incentivise a reduction, or to either subsidise or cut the import excise on other ‘healthier’ vegetable oils.

### Global evidence: systems for administering taxes

Third, objects of the tax should be clearly identifiable within a country’s tax system,^21^ ideally using existing mechanisms for differentiating between food commodities. Clear and consistent food labelling is critical to be able to define and categorise foods. Most countries have adopted the CODEX Standard for Labelling of Prepackaged Foods, mandating the declaration of all ingredients on the food label, together with the display of nutrient composition against nutrients protein, carbohydrate, total fat and energy. However, the variability in the display of other nutrients associated with NCD risk (including sodium, sugar, saturated fat and trans fatty acids) has meant that nutrient-based taxes are less often applied than product specific taxes, despite being potentially more impactful.^34^ There is no current standard for labelling food according to environmental impacts, though this is likely to change.

Countries can use the international Harmonised Tariff Systems (HS codes) to identify and tag commodities for taxation, as it differentiates all food (and non-food) commodities based on their product composition, and is used by nearly all countries. However, because HS codes are unrelated to nutritional composition or manufacturing processes, using these to target ‘unhealthy foods’ or foods with negative environmental implications may invariably lead to some ‘over capture’ (the taxation of subcategories that may not be primary targets of taxation).

### Pacific context: administrative considerations for tax design

**Figure 2:**
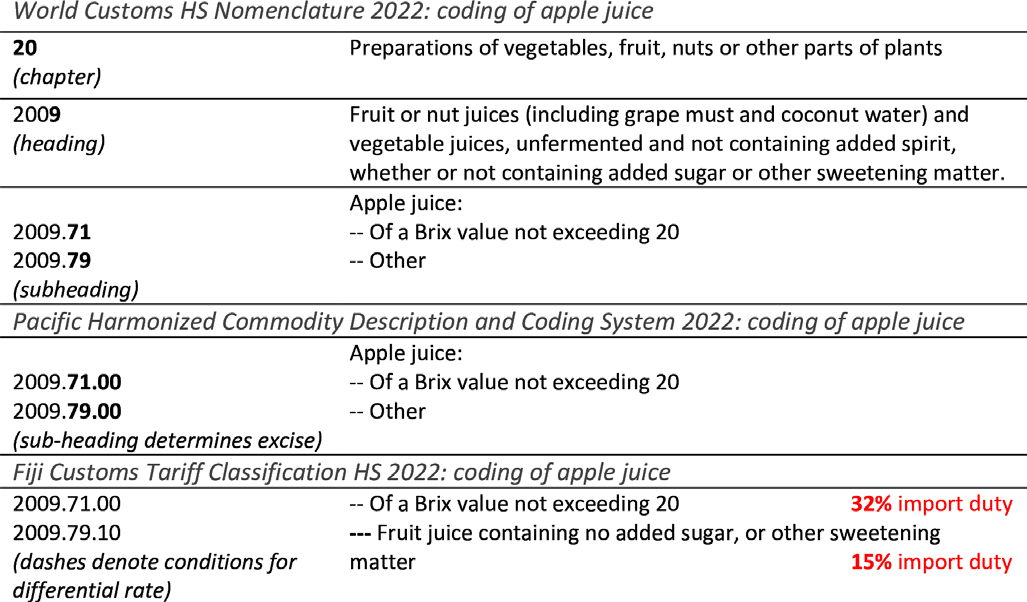
HS commodity nomenclature: example of apple juice. HS, Harmonised Tariff Systems.

Administrative systems for delivery of excises depend on the tax target. In PICs, nutrient-based, ingredient-based and food-based taxes can all be administered against HS coding systems, given these are already widely used and contain some commodity description. Countries can avoid ‘overcapture’ by performing a national ‘split’ of HS codes to demarcate commodities with specific characteristics (ie, based on nutrient contents, across a tier of values), and to signal their import conditions (eg, a 20% tax). PIC’s often denote HS splits with a series of dashes ‘---’, as in Fiji in figure 2. Any taxes linked to nutrient thresholds or specific ingredients require review of food regulations to ensure that the relevant nutrient and ingredient information is mandated for display on food labels in those settings. For example, the 2017 Samoa Food (Safety and Quality) Regulations mandate the display of energy, protein, fat, sodium and carbohydrate, but not sugar, saturated fat or trans fatty acids. Thus, a tax against sodium contents would be more straightforward for Samoa to implement than a tax on sugar.

The selection of HS codes against which to assign specific tax conditions would be strengthened with oversight under a national expert food tax committee comprised of nutrition and/or environmental experts, together with representatives from health, agriculture and finance. The new Pacific Nutrient Database, containing nutrient composition data for 22 nutrients in 800 Pacific foods, could be used to examine the nutrient contents of basic commodities.^35^ Regional collaboration on nutrient-based HS ‘splits’ could facilitate harmonisation in food taxation across the region, increasing purchasing power to encourage reformulation or product substitution.

New HS code-based excises can be communicated via updated tariff schedules, customs brokers and in ASYCUDA, the computerised customs system managing foreign trade and excise procedures, already applied in 15 PICS (a figure that is growing). Postclearance audits would help to ensure importers were complying with new HS coding conditions, where there is capacity to do so.

## Consideration 2: setting the tax base, rate and mechanism

### Global evidence: setting the base, mechanism and rate

As a fourth stage, countries need to determine taxation base, mechanism and rate. Mechanisms for taxes and subsidies can be applied based on price (ad valorem) or volume (including nutrient content). Ad valorem taxes are applied as a percentage of the existing price, which can make them more straightforward to administer, but they can have a disproportionate impact on the more expensive products, leading to substitution with cheaper brands.^21^ Volumetric taxes applied by weight can also be applied against specific characteristics of a product; for example, using the classification systems outlined in table 2. Food taxes can also be tiered against nutrient contents, or other easily identifiable characteristics, to incentivise manufacturers to reduce use of inputs linked to NCDs or with negative environmental implications.^5^

Excise taxes are a reliable mechanism as they are applied at importation and production stages, minimising the number of entities from which the tax needs to be collected, keeping administrative costs low^5^ and maintaining compliance with trade agreements.^21^ In comparison, sales taxes are applied at point of sale, meaning that shelf prices may not reflect the tax, and incentivise behaviour change. Sales taxes could be useful in some contexts at the jurisdictional level in lieu of federal excise laws (eg, council or state level initiatives), but they are less reliable where there is a significant informal market.

The tax rate needs to take into account the potential incentive created by different rates, in line with the objectives of the tax. Small taxes (ie, <10%) are unlikely to substantially impact food consumption,^11 36 37^ and thus raise revenue more consistently. Larger taxes (20% or more of the price) that are indexed to inflation are more likely to reduce consumption.^25 37^

### Pacific context: setting the base, mechanisms and rate

Food taxes that conform with existing mechanisms for taxation will be more straightforward to implement. Mechanisms that have proven feasible in Pacific countries include import tariffs (used across a range of commodities), manufacturer excises (alcohol) and licensing fees (tobacco), all of which are reflected in the final shelf price.^17^ To address concerns regarding discrimination in tax design, countries will need to ensure that taxes are applied using the same criteria at both import and production stages.^24^ Over half of all PICs have now applied manufacturers excises on SSBs, and extending these to food offers an incentive for policy-makers to prioritise strategies to improve challenges with regard to compliance of self-reported manufacturers excise.^15^

Pacific countries are applying a mix of volumetric taxes and ad valorem taxes (see online supplemental material), which indicates that a variety of base and tax mechanisms are feasible for food taxation.^16^ Countries opting for a nutrient-based system for food taxation could adopt a tiered tax based on specific nutrient thresholds by setting up such import conditions in ASYCUDA, requiring exporting countries to declare specific characteristics (eg, tier of tax) for specific treatment. Import and revenue authorities would benefit from a good understanding of food composition and food labelling, enabling them to undertake effective compliance checks.

Some PICs have already implemented quite high tax rates (see online supplemental material),^16^ modelling close to best-practice recommendations with regard to tax effectiveness.^11^ For instance, Tonga in 2013 and 2020 adopted a series of taxes on unhealthy foods of around 15%, including a tax tiered against the sugar content in SSBs, with SSBs containing the highest amount of sugar taxed at US$1.80/L.^30^

## Consideration 3: tax design that addresses fiscal and political dynamics

### Global evidence: improving political and public support for the tax

A final consideration is acceptability of food taxation, which is shaped by policy framing, narratives and design. In the context of the current health and economic crises, it is important to note that health taxes (food, tobacco and alcohol) could come close to funding half of the revenue shortfall being faced by LMICs resulting from COVID-19-related economic downturn.^5 38^ Revenue ‘earmarking’, where revenues from taxes are directed towards addressing other population health interventions^17 37^ could be used where the delivery of universal healthcare is dependent on increasing government resources.^37^ Integrating into tax design some commitments to adopt complementary policies including subsidies on healthier foods for low-income earners can help to address equity concerns. Food industry actors have consistently opposed food taxes on the basis that they will exacerbate economic downturns and unemployment.^39^ However, health and finance collaboration can ensure tax design addresses concerns for the impacts of food taxes on employment, economics and equity concerns,^5^ including higher price sensitivity among lower-income groups.^37^

### Pacific context: improving political and public support for the tax

PICs face massive and long-term economic regression as a result of the economic impacts of the COVID-19 pandemic,^20^ providing a strong basis for PICs, already highly motivated by the NCD crisis, to adopt policy framing centred on the potential of food taxes as a post-COVID-19 recovery strategy that has a dual-benefit of averting a pipeline of health spending (table 3).

**Table 3:** Options for addressing fiscal and political dynamics

Evidence to improve political palatability	Implications for tax design in the Pacific Islands context
Impacts on revenue	
Evidence suggests that health taxes applied to tobacco, alcohol and food could come close to funding up to half of the revenue shortfalls associated with COVID-19 in LMICs^38^	Consumption data can be used to estimate potential revenue and health impacts associated with specific pricing policies (taxes and subsidies).^15^ Taxes can be complemented with commitments to increase resources for policies that improve healthy food access to vulnerable groups (eg, children in schools).
Impacts on industry	
Evidence suggests that impacts on industry have been conflated and that short-term losses experienced by industry are largely offset by consumer reallocation of spending to other goods and services in the medium- to long-term.^39^ Overall, there is no robust evidence to support industries’ argument or employment loss; rigorous studies show net minimal loss and even slight increases in employment.^39^	Losses to industry profits are likely to be averted by shifting of purchases towards locally produced foods and beverages, or other goods and services.^39^
Equity considerations	
Higher price-sensitivity among lower-income population groups has led to concerns that health taxes may be regressive, with a greater impact on lower-income consumers.^37^ However, taxes can be considered a strategy for addressing inequities in consumption and poor health^13 37^ because low-income earners are more responsive to price subsidy.^11^	Accompanying unhealthy food taxes with subsidies on healthier foods could ensure that pricing policies are made more equitable and impactful for low-income earners.
Earmarking	
Revenue earmarking for health promotion is being used in 33 countries.^5^ Concerns around budget rigidity and fungibility can be allayed with more informal earmarking approaches.	Advocates can integrate into tax design commitments to fund complimentary nutrition policies, or committees to oversee additional prevention spending.
Policy coherence	
Greater coherence with nutrition policy would help to overcome capacity limitations by creating efficiencies for implementation.^5^	Implementation is more straightforward where food regulations specify display of target nutrients on nutrition labels.^15^ Reviewing legislated price protections would enable updates that are coherent with objectives of food taxes.

LMICs, low-income and middle-income countries.

Revenue earmarking has proven to be administratively challenging for some Pacific countries due to the absorption capacity.^17^ However, Pacific countries can allay concerns around equity impacts by committing equivalent resources to a range of policies to address food access and affordability, for example, the provision of clean drinking water and free fruit and vegetables in schools. This will to an extent overshadow claims of economic downturn and employment loss previously purported by food industry. Food companies have played a role in downplaying food taxes in PICs, however very little unhealthy food trade is produced in Pacific countries.^33^

## Coherent policy design for food taxes

Creating an enabling policy environment involves ensuring that government policies are coherent with health and environment objectives and can enable effective implementation of food taxes. Many countries have existing food pricing policies, including subsidies and price controls, and it is common to see different import excises being applied across different food commodities for reasons unrelated to health. Reviewing these policies for consistency with national health objectives will be important to ensure that countries avoid undermining health taxes, for example by ensuring that foods with known NCD risk (eg, table salt) are not being price protected or promoted, and by considering parallel adoption of a range of complementary policies (eg, subsidies on fruit and vegetables).^11^

## Conclusions

This piece has examined the global evidence for food taxes with a view to identifying practical lessons for policy design, adoption and implementation, using the Pacific Islands as a case study. PICs have both the commitment and imperative to progress policies to address relative affordability of healthy and unhealthy foods. This analysis provides a tool to support Pacific countries in the design and implementation of food taxation policies that draws on Pacific-generated evidence, and adopts a Pacific conceptualisation of healthy diets. Applying taxes that comply with existing tax systems will improve feasibility and impact (table 4). This analysis highlights the feasibility of food taxation in one context, and a range of options for policy-makers to frame food taxes in a way that promotes a win-win-win across economies, individuals and the environment, aligning to regional food systems objectives. However, accompanying food taxes with subsidies on healthier alternatives with a lower environmental impact will make food taxes more equitable and impactful.

**Table 4:** Summary of policy options for food taxation in Pacific Islands

Consideration	Principles	Policy options, using the Pacific Island region as a case study
Identifying the objects of taxation	Pricing policies must be contextually relevant Pricing policies must be administratively feasible within the tax system	Using ‘Foods to Avoid’ in the Pacific Guide to Healthy Living as a basis for identifying ‘unhealthy’ food categories in the Harmonised Tarif System (HS).
		Splicing food commodity HS level 8 codes to generate sub-headings and regional subheadings against nutrient threshold for sugar, sodium or fat.
	Pacific countries are promoting sustainable food systems	Using ‘Foods to Avoid’ in the Pacific Guide to Healthy Living as the only available environmental indicator.
Setting the rate and mechanisms	Mechanism must be feasible within the tax systems	Applying volumetric excises to HS codes that are comprised of foods with NCD risk.
	Pricing aligns with global evidence on effectiveness	20% or more price increase 15% or more price subsidy
Fiscal and political dynamics	Address issues surrounding political palatability	Analysing household income and expenditure surveys to demonstrate revenue and consumption benefits.
	Pricing policies must be coherent with other government priorities	Food taxes can be countered with fruit and vegetable subsidies for low-income groups, with support for local businesses producing healthy and sustainable foods.
		Food regulations can be updated to ensure that back-of-pack food labels display all requisite nutrient contents.
		Other fiscal policies (eg, price protections, import levies, subsidies) can be reviewed for alignment with pricing policy aims.

NCD, non-communicable disease.

## Data Availability

All data relevant to the study are included in the article or uploaded as online supplemental information.
