# Supplementary material for: Scaling up food pricing policies in the Pacific: a guide to action
Source: BMJ Glob Health. 2023 Oct 9;8(Suppl 8):e012041. doi: 10.1136/bmjgh-2023-012041 (PMC10565307; doi:10.1136/bmjgh-2023-012041)
Supplement: Supplementary data [file bmjgh-2023-012041supp001.pdf]

Supplementary material

	Examples of food taxes already legislated in Pacific countries
**Cook Islands**	*Excise Duties Table 2018* (by weight)<br>• Water, including mineral waters and aerated waters, containing added sugar or other sweetening matter or flavoured, and other non-alcoholic beverages, not including fruit or vegetable juices: $9.37/kg
**Kiribati**	*Excise Tax Act 2013* (by weight)<br>• Prepared or preserved mackerel: 50%/kg<br>• Chocolate and other food preparations containing cocoa: 50%/kg<br>• Beet sugar, cane sugar: 20%/kg<br>• Prepared or preserved meat, meat offal or blood (of swine e.g. tinned ham): 15%/kg<br>*Excise Tax Act 2013* (by volume)<br>• Water, including mineral waters and aerated waters, containing added sugar or other sweetening matter or flavoured, and other non-alcoholic beverages, not including fruit or vegetable juices (40%)
**Samoa**	*Customs Tariff Act (Amended) 2019 – First Schedule* (by percentage)<br>• Chocolate and other food preparations containing cocoa: 8%<br>• Instant noodles: 8%<br>• Crispbread, gingerbread, sweet biscuits, rusks, toasted bread, pastries, cakes, pancakes and crisp savoury food products: 8%
**Tonga**	*Excise Tax (Amendment) Order 2013* (by weight)<br>• Lard and other pig fat: $1/kg<br>• Tallow: $1/kg<br>Excise Tax (Amendment Order) 2018 (by weight)<br>• Butter: $1.50/kg<br>• Dairy spreads: $1.50/kg<br>• Turkey tail: $2.00/kg
**Tuvalu**	*Customs Excise Duties Order 2020* (by percentage)<br>• Twisties: 50%<br>• Milk chocolate: 45%<br>• Brown sugar, Pork Luncheon, Spam: 35%<br>• Salted beef, frankfurts, condensed milk: 30%<br>• Non-fortified noodles: 25%<br>• Corned beef: 20%<br>• Turkey tail, lamb flaps and necks: 15%<br>• Tin fish (e.g. Mackerel, Sea King), canned fruits: 10%<br>• Ice-cream: 5%
